# Mucosa-associated but not luminal *Escherichia coli* is augmented in Crohn’s disease and ulcerative colitis

**DOI:** 10.1186/1757-4749-4-21

**Published:** 2012-12-13

**Authors:** Helton Luis de Souza, Vanessa R de Carvalho, Fernando Gomes Romeiro, Ligia Yukie Sassaki, Rogeria Keller, Josias Rodrigues

**Affiliations:** 1 Distrito de Rubião Junior, CEP 18618–970 Botucatu SP Brazil, Laboratory of Medical Bacteriology, Department of Microbiology and Immunology, Institute of Biosciences, State University of São Paulo (UNESP), Sao Paulo, Brazil; 2 Distrito de Rubião Junior, CEP 18618–970 Botucatu SP Brazil, Department of Internal Medicine, Botucatu Medical School, State University of São Paulo (UNESP), Sao Paulo, Brazil

**Keywords:** *Escherichia coli*, Bacteria, Virulence, Crohn’s disease, Ulcerative colitis

## Abstract

**Background:**

*Escherichia coli* is believed to participate in the etiology of Crohn’s disease (CD) and possibly of ulcerative colitis (UC), due at least in part to the observed rise in the number of these bacteria in the gut microbiota of CD and UC patients. Nevertheless, it is not fully understood whether this quantitative variation occurs equally throughout the mucosal and luminal spaces of the gut. To assess this question, stools and mucosa biopsies from distinct intestinal sites were cultured aiming at determining their *E. coli* concentration. The cultures were additionally screened for the presence of some virulence genes of pathogenic *E. coli*.

**Results:**

Analyses of clinical materials from 14 controls (38 biopsies and 14 stools samples), 11 CD (25 biopsies and 11 stools samples) and 7 UC patients (18 biopsies and 7 stools samples) indicated no significant variation in the number of *E. coli* present in stools, but a rise of at least one log_10_ CFU/mg in biopsies from the ileum of CD patients and the sigmoid and rectum of CD and UC patients. The cultures were screened for the presence of *E. coli* attaching and effacing (*eae*), invasion plasmid antigen H (*ipaH*), aggregative adherence transcriptional activator (*aggR*), Shiga cytotoxins (*stx*), and heat labile enterotoxin (*elt*) and the following serine proteases autotransporters of *Enterobacteriaceae* (SPATE) genes: plasmid encoded toxin (*pet*)*,* secreted autotransporter toxin (*sat*)*, Shigella* extracellular protein (*sepA*)*,* protein involved in intestinal colonization (*pic*) and *Shigella* IgA-like protease homolog (*sigA*)*.* Six of the 10 genes screened were detected in the total of samples investigated: *aggR, eae, pet, sat, sepA* and *sigA*. No difference in the prevalence of any of these markers was observed in cultures from different clinical materials or groups of patients.

**Methods:**

Bacterial quantitation was carried out following cultures of diluted samples suspensions in MacConkey agar, Wilkins Chalgren agar for anaerobes, *E. coli/*coliform chromocult agar, and blood agar. Screening for *E. coli* virulence genes was performed by multiplex PCR of DNA purified from total MacConkey undiluted broth cultures.

**Conclusion:**

In CD and UC patients only the mucosa associated population of *E. coli* is augmented and the proliferation is prominent in the ileum of CD and rectum and sigmoid of both UC and CD patients which are sites where the lesions usually are observed. The augmented *E. coli* population in these sites presented a low number of the virulence markers, possibly meaning that they are not relevant for the disease process.

## Background

Intestinal microbiota is one of the many factors associated with the cause or complications of the symptoms of inflammatory bowel diseases (IBD). Microbial participation might result from dysbiosis, the action of a particular pathogen, or both. As a dominant facultative aerobe of the colon, *Escherichia coli* has long been considered among the candidate IBD pathogens. Results reported thus far [1-3] indicate that IBD patients experience a rise in the population of these bacteria. Yet, due to methodological variations regarding the source of the samples (stools vs. mucosa), quantitation method (culture dependent vs. independent), and histopathological activity of the sampled tissue (inflamed vs. no inflamed), the conclusions of different works are not always coincident. Some reports indicate an increase in mucosa-associated *E. coli* population only in Crohn’s disease (CD) [1]; others sustain that this elevation is also observed in ulcerative colitis (UC) [3]. The contribution of mucosa-associated bacteria is more relevant than the lumen residing bacteria to the formation of mucosal lesions [4]. While cultures of mucus depleted colonic biopsies from controls are sterile, cultures under the same treatment of corresponding biopsies taken from CD patients show a high number of *E. coli*[1]. Rise in mucosa-associated *E. coli* from CD patients has also been demonstrated in an immunohistological study using polyclonal antibodies directed to *E. coli* antigens [5]. Analyses by temperature gradient gel electrophoresis of 16SrDNA/RNA amplicons from fecal bacteria of UC patients demonstrated a lower bacterial diversity associated with not only the dominance but with the activity of *E. coli*[2]. The proliferation of *E. coli* in a given gut mucosal site seems not to correlate with the occurrence of inflammation and the exceeding *E. coli* carries few or no traditional virulence markers [3]. Differences seem to exist regarding the ability of *E. coli* isolates from CD and UC cases in triggering the processes responsible for the characteristic lesions of each of these IBD. According to Rhodes [4] in CD, the bacteria enter the lymphoid tissue, via M cells, persist within regional macrophages leading to granulomatous inflammation, which underlies clinical manifestations, such as ulcerations, obstructions and fistulas. Around 36-40% of *E. coli* isolates from CD patients show the ability to invade epithelial cells [6,7] and the virulence potential of the adherent and invasive *E. coli* (AIEC) is well recognized [8]. In UC, flagellin mediated bacteria interaction with TLR-5 on mucosa cell surface prompts IL-8 release and ensuing neutrophil recruitment and activation [4]. The flagellin access to cell basolateral exposed TLR-5 is thought to be facilitated by the abnormally permeable mucosa seen in UC. Recent work reported a high prevalence, in rectal biopsies of UC patients, of enteroaggregative adherent *E. coli* (EAEC) [9], a typically non-invasive pathovar, whose virulence attributes include the induction of mucus secretion by goblet cells, of IL-8 release by mucosal cells, and biofilm formation [10] – features that could indicate an eventual role for these bacteria in the pathogenesis of UC. Most of studies aiming at determining variation in the concentration of *E. coli* in IBD analyses luminal or mucosa associated bacteria. In the present work, by investigating stools and mucosa biopsies samples from distinct gut sites, we show that while no quantitative case–control variation is observed in *E. coli* population from stools, the number of these bacteria residing in particular gut mucosal sites of IBD patients, notably those where the lesions often concentrate, is increased.

## Results

### Bacterial quantitative variation

**Figure 1:**
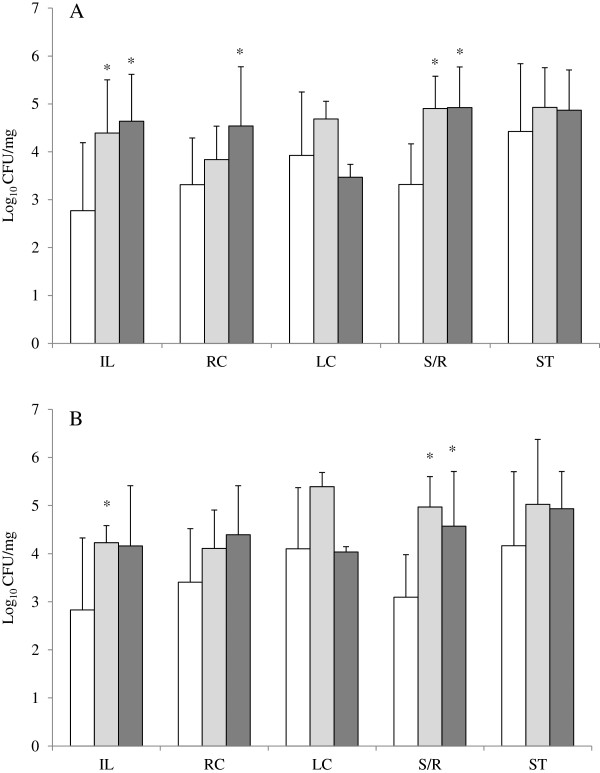
**Average bacterial concentration (CFU/mg) in distinct clinical samples from Controls (white bars), Crohn’s Disease (grey bars) and ulcerative colitis (dark bars) patients.A**, Total counting in MacConkey agar cultures; **B**, Purple colonies counting in brilliance *E. coli/*coliform agar cultures. IL, RC, LC, S/R, ST, respectively, biopsies from ileum, right colon, left colon, sigmoid and rectum and stools. Asterisks indicate values with significant differences (*P<*0.05, Paired *t-*test) in comparisons with controls. See Table 2 for numbers of samples analysed per clinical material.

Bacterial quantitation was performed by counting of colonies grown in cultures seeded with samples suspensions, and expressed as log_10_ of the number of colonies forming units per milligram (CFU/mg). The suspensions were cultured in aerobic conditions in MacConkey agar, chromocult for coliforms agar and blood agar, and anaerobically in Wilkins-Chalgren agar. When compared with controls, although most samples from both CD and UC showed higher bacteria numbers in the four culture media employed, statistically significant differences were observed only for biopsies samples, but not for stools cultured in any of media, for either of the IBD patients groups. By comparison of cultures from sigmoid and rectum (S/R) biopsies of CD patients with controls in all media tested, the number of CFU for biopsies of the CD patients was at least of one log_10_ unit higher. Superior bacterial concentration of this same magnitude was also seen for S/R biopsies from UC patients in MacConkey, chromocult and Wilkins-Chalgren agar cultures. The same was observed for ileum biopsies from both UC and CD patients and for right colon biopsies from UC patients in MacConkey agar cultures (Figure 1A). Counting results in chromocult agar revealed no significant differences in the number of non-*E. coli* coliforms (green and red colonies) for biopsies of any gut site from both UC and CD. However, positive variation in the number of *E. coli* (purple colonies) was ca. 1.5 and 2.0 log_10_ units, respectively for ileum and S/R biopsies from CD patients and ca. 1.5 log_10_ units for S/R biopsies of UC patients (Figure 1B). The *E. coli* identity of purple colonies from chromocult cultures of every clinical sample was checked by biochemical tests. Fewer than 2% of these colonies had not their *E. coli* identity confirmed.

### Virulence genes

**Figure 2:**
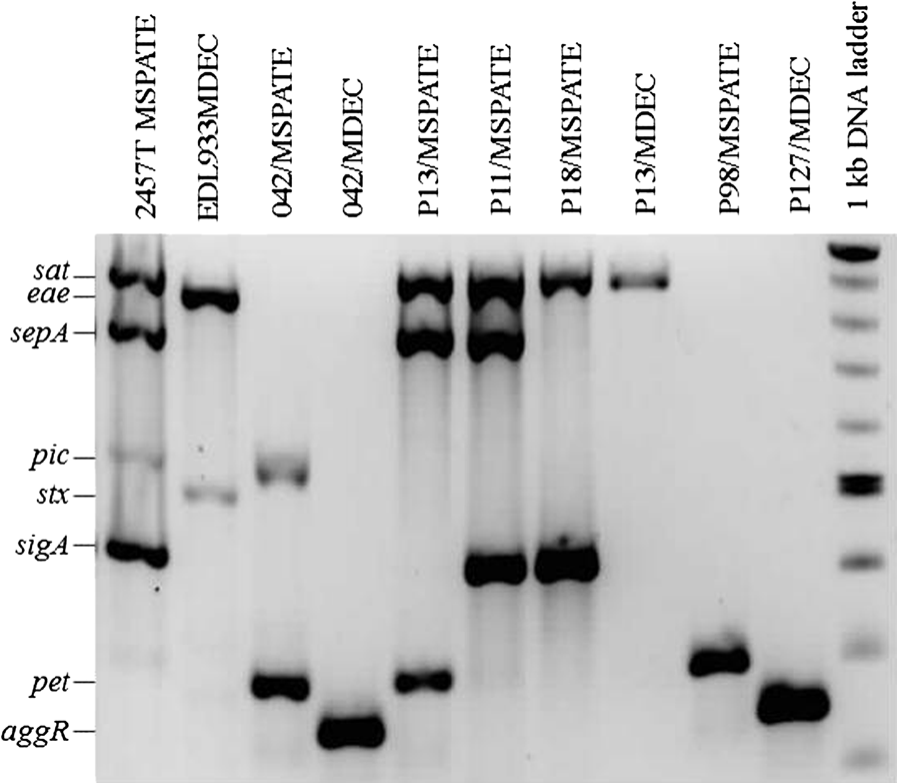
**DNA amplified by multiplex PCR, with primers for Diarrheagenic *E.coli *typical virulence genes (MDEC), and serine protease autotransporters of *Enterobacteriaceae *(SPATE) genes (MSPATE).** Lanes 1–4, Reference strains; lanes 5–10, Pools of Gram negative bacteria (growth in MacConkey broth) from some clinical samples (see Table 1). Lane 11, 1kb DNA ladder.

MacConkey broth cultures from 67 distinct clinical samples (26 from controls; 25 from CD and 16 from UC patients) were submitted to two independent multiplex PCRs – one for presence of Diarrheagenic *E. coli* (DEC) typical virulence genes [*E. coli* attaching and effacing (*eae*), invasion plasmid antigen H (*ipaH*), aggregative adherence transcriptional activator (*aggR*), Shiga cytotoxins (*stx*), and heat labile enterotoxin (*elt*)] and other for the following serine proteases autotransporters of *Enterobacteriaceae* (SPATE) genes: plasmid encoded toxin (*pet*)*,* secreted autotransporter toxin (*sat*)*, Shigella* extracellular protein (*sepA*)*,* protein involved in intestinal colonization (*pic*) and *Shigella* IgA-like protease homolog (*sigA*)*.* Six of the 10 genes screened were detected in the total of samples investigated (Figure 2 and Table 1): *aggR, eae, pet, sat, sepA* and *sigA.* With the exception of *pet*, which was detected only in controls, all of the remaining genes were found in samples from both IBD and control patients. The most common genetic markers detected were the SPATEs *sat* and *sigA* genes*.* There was no statistically significant difference in the prevalence of any of the genes searched for in comparisons between cases and controls for a given clinical sample, or between distinct clinical samples of a given subject.

**Table 1:** Number of virulence genes detected in MacConkey broth cultures of distinct clinical samples from each groups of patients

**Virulence gene**	**Clinical sample^a,b^**														
	**Ileum**			**Right colon**			**Left colon**			**Rectum/sigmoid**			**Stools**		
	CO (4)	CD (6)	UC (3)	CO (6)	CD (5)	UC (4)	CO (5)	CD (2)	UC (2)	CO (3)	CD (6)	UC (3)	CO (8)	CD (6)	UC (4)
*aggR*	1			1	1		1						1		
*eae*	1	1		1						1	1				
*pet*	1												1		
*sat*	1	1	1	3	2	3	1	1		1	1	1	3		1
*sepA*	1			1	1										
*sigA*	1	2	1	2	2	3	1	1			3	1	2	3	1

^a^ CO, Controls; CD, Crohn’s Disease; UC, Ulcerative Colitis.

^b^ ( ), Number of samples tested.

## Discussion

As an indigenous organism of the gut, *E. coli* was for long considered unsuspected of causing intestinal diseases [11] but over last decades, following the description of multiple enteric *E. coli* pathovars and their epidemiological association with enteritis, it became one of the most relevant human and animal bacterial enteropathogens, associated with several gastrointestinal diseases ranging from food poisoning to different clinical manifestations of diarrhea [12]. Accumulated evidences lend support to its involvement with the etiology of IBD as well, in particular with CD, wherein AIEC is thought to be able to invade regional macrophages leading to the formation of the epithelioid granulomas [8] typically found in histological preparations of gut biopsies from CD patients. Elevation in the number of *E. coli* in the gut of IBD patients [13] and in serum antibodies titers against some of its antigens [14,15] are among the first clues of its role in this disease. Rise in *E. coli* number was demonstrated in stools [2] and mucosa of distinct gut sites [1,3,16]. The increase in *E. coli* number in IBD patients has also been correlated with disease activity [13].

To get a broad picture of the quantitative variation in *E. coli* population from IBD patients, we have analysed cultures on *E. coli/*coliform agar of samples from at least two distinct clinical materials per patient, consisting of stools and/or biopsies of different gut sites of controls and individuals diagnosed with CD and UC. Statistically significant case-controls differences in the number of bacteria were observed only in *E. coli* counting for cultures derived from particular mucosal sites, namely ileum of CD and sigmoid and rectum of both CD and UC patients, which showed a higher bacterial concentration, in comparisons with corresponding sites of controls. In contrast to previous observations [3], the increase in *E. coli* number reported here was not accompanied by a rise in the number of non-*E. coli* coliforms.

MacConkey broth cultures from sample suspensions of each clinical material were PCR screened for ten virulence genes, five of which recognized as markers for Diarrheagenic *E. coli* (DEC) detection [12,17], and five of them corresponding to the SPATE genes *pet, sat, sigA, pic* and *sepA.* Of the five DEC genes screened, only *eae* and *aggR* were detected, in 5 of the 67 samples tested (Table 1). The low prevalence of DEC genes in cultures of these IBD clinical samples are not too different from the results of other investigators, who screening bacterial isolates, found no *E. coli* positive for any of these markers [3,16]. Also, the low prevalence of these classical DEC virulence genes in IBD clinical samples is in agreement with previous observations arguing that IBD *E. coli* are more closely related to extra intestinal pathogenic *E. coli* (ExPEC) [3,4].

The reasoning for the search of SPATE genes among IBD clinical samples took in account not only the wide distribution of SPATE among DEC and ExPEC isolates [18] but also the variety of biological activities of these proteases [19], which could eventually play some role in the pathogenesis of IBD. Furthermore, EAEC*,* a pathovar showing high prevalence of SPATE genes [20] were recently found to be dominant among IBD *E. coli* isolates [9]. With the exception of *pic,* all the remaining of the five SPATE genes searched for were found among the samples screened, with *sat* and *sigA* identified in nearly all of them. Nevertheless, no statistically significant differences could be observed in the prevalence of any of the SPATE genes identified (*pet, sat, sepA* and *sigA*) among the different clinical samples or groups of patients investigated. In the work by Kotlowski et al. [3], where a PCR screening for 8 SPATE genes was carried out, a higher prevalence of SPATE positive *E. coli* were found among isolates from IBD patients as compared to controls. Since in their analyses they used pure *E. coli* cultures instead of total Gram negative cultures from the samples, the nature of the PCR target as well as the number of genes searched for might explain the observed divergence in relation to our results.

The proliferation of *E. coli* raises the question whether the exceeding bacteria plays an active or secondary role in IBD etiopathogenesis. The prevalence of haemolytic and necrotoxic *E. coli* strains has been shown to be higher in patients in relapsing UC, but follow-up analyses demonstrated that these bacteria tended to follow rather than precede the onset of relapse attack [13]. On the other hand, AIEC has being detected in proportion as high in early as in chronic ileal lesions of CD patients [6]. It is believed that in CD, the bacterial adhesion is favoured by abnormal receptors exposition in cell surface of genetically susceptible hosts [8]. Soluble plant fibers, to which a therapeutic potential in treatment of CD has been attributed, are able to inhibit this bacterial adhesion [1].

Although previous report suggests an active rather than secondary role for *E. coli* in IBD, on the basis of a high prevalence of strains possessing virulence markers in IBD patients [3], this indication is not supported by the data of the present work. By using as PCR target gross (total) Gram negative bacteria cultures which are more representative than a few colonies of the bacterial population in the samples, we were unable to detect difference in the prevalence of the *E. coli* virulence genes searched for among distinct groups of patients or clinical material investigated here. The genes consisted of five markers traditionally used for identification of DEC and five SPATEs genes, categories of proteases which up to date were not found in non-pathogenic organisms [18]. Moreover, the bacteria proliferation in IBD patients was noticed in sites where the lesions are prominent and usual, namely the ileum, sigmoid and rectum in CD and the sigmoid and rectum in UC. The apparently non-random *E. coli* abundance in these sites and its unaltered number in stools do not preclude the possibility that they represent secondary local colonizers. Also, in view of their localization, luminal bacteria could be a more direct target for drugs interfering with bacterial species composition of the gut, and thus the history of drugs intake could explain why the increment in the number of mucosa colonizing *E. coli* was not also observed for luminal population of these bacteria, among IBD patients. Despite of the low number of genes searched for and the restricted quantity of some samples, such as left side colon, the uniform distribution of virulence genes among the various clinical materials in controls and IBD patients might indicate an irrelevance of these factors and/or the *E. coli* pathovars marked by them for the pathogenesis of IBD.

There are numerous evidences to support the involvement of *E. coli* with CD and hence AIEC is recognized as new pathovar within the species [21], but the link of *E. coli* with UC is not as clear. We speculate that in UC, and eventually in CD, aggregative adherent *E. coli* might play some role, at least in aggravating already established inflammatory processes. This group of bacteria, which is extremely heterogeneous in regard to expression of virulence factors and pathogenic potential, is classified in typical and atypical EAEC, based upon the presence of the *aggR* transcriptional activator of virulence genes in the first and absence in the last [21]. Atypical EAEC includes commensal, intestinal and extra-intestinal pathogenic *E. coli.* Testing some *E. coli* isolates derived from the Gram negative cultures studied here for the ability to adhere to Hep-2 indicated the presence of atypical EAEC in samples from IBD cases (data not shown). With the characterization of these bacteria presently under way in our laboratory we hope to get relevant information which will be of help in understanding their proliferation in the gut mucosa of IBD patients.

## Conclusion

Analyses of samples from stools and biopsies of different intestinal mucosa sites from controls and patients diagnosed with CD and UC indicate that while the number of *E. coli* is unaltered in the stools, the amount of these bacteria is higher in ileal biopsies from CD and in the sigmoid and rectum of both CD and UC patients. Tests for DEC virulence markers and some SPATE genes did not show any difference in the prevalence of these genes in the augmented *E. coli* population. The elevation of these bacteria in sites where the lesions usually appear seems to indicate that their local establishment is secondary to the initiation of the inflammatory processes.

## Methods

### Patients and clinical samples

The study groups comprised of subjects attending the UNESP University Hospital, Botucatu SP Brazil, for routine colonoscopy who fulfilled the following inclusion criteria: patients not under antibiotic therapy and not having history of infectious intestinal diseases for at least 2 months before the examination and who agreed, by signing a Consent Form, with the destination of part of their clinical materials for research purposes. All the procedures regarding the approaching of the patients were approved by the local Committee on Ethics in Research. A number of 11 subjects diagnosed with CD, 7 with ulcerative colitis (UC), and 14 Controls were investigated. The IBD diagnosis was based on clinical, colonoscopic and histopathological criteria [22,23]. The controls group comprised of individuals who not fulfilled the diagnosis criteria for IBD, showing no detectable intestinal pathology or intestinal bleeding of non IBD or non-infectious origin. Clinical materials included stools and mucosal biopsies from the ileum and different parts of the large intestine (Table 2). Most of biopsies were sampled from sites showing no detectable or mild endoscopic activity. A number of at least two distinct clinical samples, including stools and biopsies, from each patient were taken (Table 2). After collection, the stools were maintained in refrigerator for ca. 72 h before preparation for bacterial cultures.

**Table 2:** Number of mucosal biopsies and stools analysed per group of patients

**Clinical sample**	**Groups^a,b^**		
	**CO (14)**	**CD (11)**	**UC (7)**
Ileum	7	6	5
Right colon	10	7	6
Left Colon	11	3	3
Rectum/sigmoid	10	9	4
Stools	14	11	7

^a^ CO, Controls; CD, Crohn’s Disease; UC, Ulcerative Colitis.

^b^ ( ), Numbers of patients.

### Bacterial cultures

Unless otherwise indicated, all the culture media and reagents for bacterial cultures were manufactured by Oxoid (Basingstoke, Hampshire, England). Incubation temperature for all cultures below was 37°C. Samples weighting ca. 5–15 mg of freshly (within 3 h after colonoscopy) collected biopsies or refrigerated stools were vortexed in 100 μl of 0.9% sterile saline dispensed in 2 ml polypropylene tubes (Axygen, Union City, USA). Biopsies were fragmented by adding sterile sand to the tubes. Half of sample suspension was transferred to MacConkey Broth and the remaining volume was subjected to serial (decimal) dilutions for bacterial quantitation in each of the following agar media: MacConkey, brilliance *E. coli/*coliform, Wilkins-Chalgren anaerobe and nutrient agar supplied with 5% defibrinated sheep blood. Wilkins Chalgren anaerobe cultures were grown for 48 h within anaerogen O_2_-depleted sealed jar. Cultures in the other media were grown for 18–24 h under atmospheric oxygen tension. The total of bacterial colonies in each agar plate was counted and the number of UFC/mg of sample calculated according to the suspension dilution. Aliquots of 50 μl of MacConkey broth cultures were transferred to brain hearth infusion broth, incubated overnight, and the resulting growth was frozen with 15% glycerol at −80°C. These Gram negative bacteria (GNB) pools were used for *E. coli* recovery and DNA purification for PCR. The presence or absence of *E. coli* in each sample was confirmed by biochemical identification of purple colonies grown overnight in Brilliance *E. coli/*coliform agar streaked with GNB pools. A number of 5–15 colonies from these cultures were individually inoculated in MacConkey, semi-solid MILi, modified Rugai [24] and Simmons citrate agars (BBL, Cockeysville, USA) wherein the following reactions could be detected: urease, indole, and H_2_S production, lactose and glucose fermentation, gas from glucose fermentation, utilisation of sodium citrate as single carbon source, bacterial movement, lysine decarboxylation and L-tryptophan deamination. Those colonies showing positive results for indole and glucose fermentation and negative results for H_2_S, L-tryptophan deamination and urease production were considered as *E. coli*.

### Virulence genes detection

Genomic DNA was purified from overnight cultures grown in Luria Broth inoculated with 20 μl of stocked GNB pools utilising the QIAmp mini kit (Qiagen, Hilden Germany). Each bacterial pool DNA was PCR screened with primers specific for genes typically found in Diarrheagenic *E. coli* (DEC) (*eae*, *stx*, *elt, aggR,* and *ipaH*) and serine proteases autotransporters of *Enterobacteriaceae* (SPATE) encoding genes (Table 3). For SPATE genes detection, the samples were first screened by a monoplex PCR using generic primers targeting genes of common sequences [3]. SPATE+ samples were then tested by multiplex PCR using primers specific for each of the following individual SPATE genes: *pet, sat, sepA, pic* and *sigA,* (see reference [20] and Table 3). Screening for DEC genes was performed by multiplex PCR according to Toma *et al.*[17], and for individual SPATEs according to Boisen *et al.*[20]. Positive controls used in all PCR were the following *E. coli* reference strains: EDL933, for *eae* and *stx;* 40T, for *elt,* O42, for *aggR* and *pet* and EIX, for *ipaH. Shigella flexneri* 2a strain 2457T was used as positive controls for *sat, sepA, pic* and *sigA.* Negative control for the PCRs was *E. coli* K12 HB101. Strain EDL933 was gently provided by Dr. I. C. A. Scaletsky from UNIFESP Medical College, SP Brazil, and *S. flexneri* 2a 2457T by Dr J. B. Kaper from Center for Vaccine Development, University of Maryland, USA. PCR master mix components were used as commercial kits, to which the template DNA and primers were added, following manufacturer instructions. Qiagen multiplex kit (Qiagen, Valencia, USA) and NEB *Taq* 5X master mix (NEB, Hitchin, UK) were used in multiplex and monoplex PCR, respectively and the oligonucleotides were synthesized by Eurofins (Eurofins MWG Operon, Huntsville, USA). The PCRs were run in a MasterCycler ProS Thermocycler (Eppendorf, Hamburg, Germany).

**Table 3:** Virulence genes, primers and DNA target sequence sizes (amplicons) of multiplex PCRs

**Gene**	**Primers (5^′^- 3>^′^)**	**Amplicon (bp)**	**References**
*stx*	F- GAGCGAAATAATTTATATGTG	518	[17]
	R- TGATGATGGCAATTCAGTAT		
*eae*	F- CCCGAATTCGGCACAAGCATAAGC	881	[17]
	R- CCCGGATCCGTCTCGCCAGTATTCG		
*sigA*	F- CCGACTTCTCACTTTCTCCCG	430	[20]
	R- CCATCCAGCTGCATAGTGTTTG		
*sepA*	F- GCAGTGGAAATATGATGCGGC	794	[20]
	R- TTGTTCAGATCGGAGAAGAACG		
*pic*	F- ACTGGATCTTAAGGCTCAGGAT	572	[20]
	R- GACTTAATGTCACTGTTCAGCG		
*aggR*	F- GTATACACAAAAGAAGGAAGC	254	[17]
	R- ACAGAATCGTCAGCATCAGC		
*ipaH*	F- GTTCCTTGACCGCCTTTCCGATACCGTC	619	[17]
	R- GCCGGTCAGCCACCCTCTGAGAGTAC		
*elt*	F- TCTCTATGTGCATACGGAGC	322	[17]
	R- CCATACTGATTGCCGCAAT		
*pet*	F- GGCACAGAATAAAGGGGTGTTT	302	[20]
	R- CCTCTTGTTTCCACGACATAC		
*sat*	F-TCAGAAGCTCAGCGAATCATTG	930	[20]
	R-CCATTATCACCAGTAAAACGCACC		

## Competing interests

No competing interests declared.

## Authors’ contributions

HLdS and VRC worked in collection, preservation and processing of the samples for bacterial quantitation and *E. coli* detection. RK assisted HLdS in PCR technical issues. LYS got and managed the patients’ clinical information. FGR conducted the patients’ colonoscopic examinations. JR supervised laboratory work, compiled the data and wrote the manuscript. All authors read and approved the final manuscript.
